# Consumption of a light meal affects serum concentrations of one-carbon metabolites and B-vitamins. A clinical intervention study

**DOI:** 10.1017/S0007114522002446

**Published:** 2023-04-28

**Authors:** Anita Helland, Marianne Bratlie, Ingrid V. Hagen, Øivind Midttun, Arve Ulvik, Gunnar Mellgren, Per M. Ueland, Oddrun A. Gudbrandsen

**Affiliations:** 1 Dietary Protein Research Group, Department of Clinical Medicine, University of Bergen, Bergen, 5021, Norway; 2 Bevital AS, Jonas Lies Veg 87, Bergen, Norway; 3 Mohn Nutrition Research Laboratory, Department of Clinical Science, University of Bergen, Bergen, Norway; 4 Hormone Laboratory, Department of Medical Biochemistry and Pharmacology, Haukeland University Hospital, Bergen, Norway

**Keywords:** One-carbon metabolism, Homocysteine, Choline, Betaine, B-vitamins

## Abstract

The transfer of one-carbon units between molecules in metabolic pathways is essential for maintaining cellular homeostasis, but little is known about whether the circulating concentrations of metabolites involved in the one-carbon metabolism are affected by the prandial status. Epidemiological studies do not always consistently use fasting or non-fasting blood samples or may lack information on the prandial status of the study participants. Therefore, the main aim of the present study was to investigate the effects of a light breakfast on serum concentrations of selected metabolites and B-vitamins related to the one-carbon metabolism; i.e. the methionine-homocysteine cycle, the folate cycle, the choline oxidation pathway and the transsulfuration pathway. Sixty-three healthy adults (thirty-six women) with BMI ≥ 27 kg/m^2^ were included in the study. Blood was collected in the fasting state and 60 and 120 min after intake of a standardised breakfast consisting of white bread, margarine, white cheese, strawberry jam and orange juice (2218 kJ). The meal contained low amounts of choline, betaine, serine and vitamins B_2_, B_3_, B_6_, B_9_ and B_12_. Serum concentrations of total homocysteine, total cysteine, flavin mononucleotide, nicotinamide and pyridoxal 5’-phosphate were significantly decreased, and concentrations of choline, betaine, dimethylglycine, sarcosine, cystathionine and folate were significantly increased following breakfast intake (*P* < 0·05). Our findings demonstrate that the intake of a light breakfast with low nutrient content affected serum concentrations of several metabolites and B-vitamins related to the one-carbon metabolism.

The transfer of one-carbon units amid molecules within and between metabolic pathways is essential for maintaining cellular homeostasis, and this process efficiently control post-translational modifications and epigenetic, energetic and redox statuses. One-carbon units are transferred within the interlinked pathways methionine-homocysteine cycle, the folate cycle, the choline-oxidation pathway and the methylation of DNA, RNA, proteins and lipids^(1)^. Homocysteine is remethylated to methionine using methyl from either N^5^-methyl-tetrahydrofolate or betaine, thereby generating methionine as well as tetrahydrofolate or dimethylglycine, respectively. Methionine is converted to the methyl donor S-adenosylmethionine, which is transformed to S-adenosylhomocysteine after donating a methyl group. Alternatively, homocysteine may enter the transsulfuration pathway to produce cystathionine, which is further metabolised to cysteine and glutathione^(1)^.

Betaine, choline (after oxidation to betaine), glucose and amino acids including methionine, serine and glycine are quantitatively important donors for one-carbon units obtained from the diet^(1,2)^. In addition, choline-derived methyl-glycine species (sarcosine and dimethylglycine), as well as serine and glycine from endogenous sources, are important contributors to the pool of tetrahydrofolate-bound one-carbon units^(1)^. Vitamins B_2_, B_3_, B_6_, B_9_ and B_12_, which are obtained from the diet, are important cofactors for several enzymes involved in the transfer of one-carbon units^(2)^.

Disturbances in the pathways involved in the one-carbon metabolism have been associated with diseases. Among the well-known health issues related to disturbances in the one-carbon metabolism is the increased risk for neural tube defect in fetus of mothers with insufficient folate intake, and the increased risk for colorectal cancer with low folate intake^(1)^. Another well-described example is elevated concentration of homocysteine that has been associated with an increased risk of CVD^(3,4)^. The homocysteine concentration is higher in patients with vascular disease^(5,6)^ and in patients with metabolic syndrome^(7,8)^, and elevated homocysteine concentration has been shown to be causally related to increased risk of type 2 diabetes^(9)^. High homocysteine concentration is associated with low concentrations of folate and vitamin B_12_, both of which are required for the remethylation of homocysteine to methionine^(1)^. A high intake of folate reduces the risk for CVD^(10)^, and a low serum folate concentration was associated with higher incidence of insulin resistance in non-diabetic USA adults^(11)^. Also the plasma concentration of the methyl donor dimethylglycine has been associated with several traditional risk factors for coronary artery disease in patients with stable angina pectoris^(12)^, whereas a high choline concentration has been associated with the risk of long-term atrial fibrillation^(13)^, an increased risk for acute myocardial infarction in non-smokers^(14)^ and adverse cardiac events in patients with suspected acute coronary syndromes^(15)^. Methyl groups are also transferred to amino acids, and several methylated amino acids have been associated with increased risk for diseases. In patients with suspected stable angina pectoris, elevated circulating concentration of trimethyllysine is a predictor for type 2 diabetes^(16)^ and for acute myocardial infarction^(17)^. Also, an elevated concentration of asymmetric dimethylarginine is an independent risk factor for CVD^(18)^, and high circulating concentrations of both asymmetric^(19)^ and symmetric^(20)^ dimethylarginine are seen in patients with chronic kidney disease.

Epidemiological studies do not consistently use fasting or non-fasting blood samples, or may lack information about the prandial status of the study participants. Little is known about whether the circulating concentrations of metabolites and B-vitamins that participates in the one-carbon metabolism are affected by the prandial status. However, *in vitro* studies suggest that several enzymes involved in one-carbon metabolism are affected by increased concentrations of insulin and glucose, resulting in increased remethylation of homocysteine to methionine^(21)^. In addition, it has been shown that the concentration of methionine was higher while the concentration of free homocysteine was lower in plasma from healthy adults after consumption of a light breakfast^(22)^. In many trials, blood is typically sampled early in the day during working hours for practical reasons, i.e. with participants in a fasting state or after having consumed a light meal. Therefore, the main aim of the present study was to assess the effects of a light meal containing carbohydrates, proteins and fats on serum concentrations of a broad panel of one-carbon metabolites and B-vitamins involved in the one-carbon metabolism. Blood was collected from adults before, and 60 and 120 min after consumption of a standardised breakfast meal. Our hypothesis was that when the participants’ metabolic status changed from catabolic to anabolic after intake of a light meal, this would result in a lower homocysteine concentration but would also affect serum concentrations of other metabolites and B-vitamins involved in the one-carbon metabolism.

## Methods

### Participants, study setting and ethics

The subjects in the present work were participants in a study designed to investigate the metabolic effects of high intake of fish for 8 weeks. In the present paper we present analyses of the samples collected at baseline. The study population consisted of adults of Norwegian ethnic origin (Caucasian) with overweight or obesity living in Bergen, Norway. Inclusion criteria were BMI ≥ 27 kg/m^2^, fasting blood glucose ≤ 7·0 mmol/l, and age 18–69 years. Exclusion criteria were pregnancy, incompatibility with fish consumption (allergies, intolerance and/or dislike), diagnosed diabetes mellitus, heart disease or gastrointestinal disease, use of medications affecting lipid metabolism or glucose homoeostasis, use of anti-inflammatory medications, use of supplements containing *n*-3 PUFA, intentional weight loss, and large fluctuation in body weight (>3 kg) during the preceding 2 months. The study design, as well as description of study participants, study setting and protocol for study visits have previously been described in detail^(23)^. Seventy-six participants were included^(24)^, and sixty-eight participants completed the trial. Three participants were excluded (one had prediabetes and two did not comply with the protocol). For two participants, we did not have a sufficient amount of blood serum for analyses; hence, serum from sixty-three participants (thirty-six women) were included in the present study, with a geometric mean (5, 95 % CI) age of 42·8 (39·9, 45·8) years and geometric mean BMI (5, 95 % CI) of 32·9 (31·8, 34·0) kg/m^2^. All participants had serum creatinine concentration and urine albumin creatinine ratio within normal ranges^(25)^. Examinations were conducted at the Clinical Research Unit at the Haukeland University Hospital, Bergen, Norway.

The study was conducted according to the guidelines laid down in the Declaration of Helsinki, and all procedures were approved by the Regional Committee for Medical and Health Research Ethics of Western Norway (REC no.: 2011/572). Written informed consent was obtained from all participants.

Health professionals performing blood sampling and personnel conducting the laboratory analyses were all blinded to participants’ identity, and all data were analysed anonymously. The trial is registered at clinicaltrials.gov as NCT02350595.

### Protocol for study visits

Examinations and samplings were conducted in the morning after an overnight fast; intake of food or drinks except water, or use of substances containing nicotine was not permitted after 10 pm the previous day. Physical exercise and alcohol were not allowed for 24 h before the visit. Blood was drawn from an antecubital vein by inserting a cannula connected to a three-way tap for repeated measures, and the system was flushed with sterile saline (0·9 %) before and after each blood sample. Blood was collected in BD Vacutainer SST II Advance gel tubes (Becton, Dickinson and Company) for isolation of serum. The staff complied with a strict protocol for pre-analytical sample handling to ensure high sample quality. Blood samples were centrifuged after 30 min at room temperature, and serum was immediately aliquoted and frozen at –80°C until analyses. Participants provided morning urine upon arrival to the hospital, and urine samples were immediately aliquoted and frozen (–80°C).

### Intervention

After the collection of fasting blood, the participants consumed a standardised breakfast consisting of one slice of white bread with 5 g margarine and 25 g strawberry jam, one slice of white bread with 5 g margarine and 20 g white cheese and 0·30 l orange juice. The estimated contents of macronutrient and energy in the standardised breakfast were 80 g carbohydrate, 14 g protein and 16 g fat, providing a total of 2218 kJ, as calculated using ‘Mat på Data 5·1’^(26)^ and information provided by the manufacturers. The contents of vitamin B_2_ (riboflavin), vitamin B_3_ (total niacin), vitamin B_6_ (pyridoxine), vitamin B_9_ (total folate), vitamin B_12_ (cobalamin), betaine (total) and choline (total) (conducted by Eurofins Food & Feed Testing Norway AS, Moss, Norway) and contents of methionine, glycine and serine (conducted by Nofima BioLab, Bergen, Norway) in the breakfast are presented in Table 1. The breakfast was consumed within 15 min. Blood samples were collected in fasting state, as well as 60 and 120 min after the participants had consumed the standardised breakfast.

**Table 1.** Contents of vitamins B_2_, B_3_, B_6_, B_9_ and B_12_, total choline, total betaine, methionine, serine and glycine in the standardised breakfast and relevant RDA values for our study participants

Amino acids	Per breakfast serving	Recommended daily allowance for adults
Vitamin B_2_ (riboflavin)	0·09 mg	1·7 mg (men), 1·3 mg (women)*
Vitamin B_3_ (total niacin)	0·48 mg	18 NE (men), 15 NE (women)*
Vitamin B_6_ (pyridoxine)	0·08 mg	1·5 mg (men), 1·2 mg (women)*
Vitamin B_9_ (total folate)	79·6 µg	300 µg (men), 400 µg (women)*
Vitamin B_12_ (cyanocobalamin)	0·58 µg	2·0 µg (men and women)*
Choline (total)	53·8 mg	400 mg (men and women)†
Betaine (total)	30·0 mg	Not defined
Methionine	<LOD	10·4 mg/kg bodyweight (men and women)‡
Serine	0·66 g	Not defined
Glycine	<LOD	Not defined

NE: niacin equivalent.

* Nordic nutrition recommendations.

† European food safety authority.

‡ WHO/FAO/UNU joint report.

Level of detection (LOD) for amino acids is 0.10 g/100 g sample, corresponding to 0.44 g/breakfast serving.

### Analyses in serum and urine

Serum and urine concentrations of total homocysteine, methionine, total cysteine, cystathionine, glycine and serine were measured using gas chromatography combined with tandem mass spectrometry^(27)^. Free choline, betaine (N,N,N-trimethylglycine), dimethylglycine, sarcosine (N-methylglycine), asymmetric dimethylarginine, symmetric dimethylarginine, trimethyllysine, 1-methylhistidine (*π*-methylhistidine), 3-methylhistidine (τ-methylhistidine) were measured in serum and urine, and creatinine was measured in urine, using liquid chromatography combined with tandem mass spectrometry^(28)^. 1-methylhistidine and 3-methylhistidine were measured by adding ion-pairs for the analytes and isotope-labelled internal standards to the existing assay^(28)^. Vitamin B_2_ (riboflavin and flavin mononucleotide), vitamin B_3_ (nicotinic acid, nicotinamide and N^1^-methylnicotinamide) and vitamin B_6_ (pyridoxal 5’-phosphate) were analysed in serum using liquid chromatography combined with tandem mass spectrometry^(29)^. Nicotinic acid, nicotinamide and N^1^-methylnicotinamide^(30)^ with corresponding isotope labeled internal standards were added to the previously published assay^(29)^. Vitamin B_12_^(31)^ and folate^(32)^ were measured in serum by microbiological assays. All biochemical analyses were performed by Bevital AS (Bergen, Norway, http://www.bevital.no).

All serum and urine samples for each analysis were analysed for each participant in random order on the same day, and samples were not thawed previously.

Reference values for serum folate and cobalamin concentrations were according to the Department of Medical Biochemistry and Pharmacology, Haukeland University Hospital; the action limit for treatment for folate deficiency is when serum folate concentration < 10 nmol/l, and the normal range for serum cobalamin concentration is defined as 175–700 pmol/l.

### Outcome measurements

The primary outcome of the present study was to compare concentrations of the selected metabolites and B-vitamins involved in the one-carbon metabolism; the methionine-homocysteine cycle, the folate cycle, the choline oxidation pathway, and the transsulfuration pathway, in fasting serum and serum collected 60 and 120 min after intake of a standardised breakfast. The secondary outcome was to compare men and women with regard to the fasting serum concentrations and the relative changes from fasting to postprandial serum concentrations of metabolites and B-vitamins, as well as urine concentrations of relevant metabolites.

### Sample size estimation

The present study exploits biological material collected at the baseline visit in an intervention study that was designed to investigate the effects of high intake of cod or salmon on post-prandial glucose regulation after a standardised breakfast in participants with overweight or obesity^(23)^. The sample size estimation for the original study showed that it was necessary to include seventy-six participants divided into three groups to ensure that twenty participants in each group completed the trial with satisfactory compliance, with a power of 80 % and *α* of 0·05^(23)^. Since the present study is, to the best of our knowledge, the first study to investigate the effects of a light breakfast on the one-carbon metabolism in healthy adults, data on effect size were not available for sample size calculation or minimally detectable effect sizes for the present study.

### Statistical analyses

Fasting serum concentrations and urine concentrations (relative to creatinine) are presented as geometric means (5, 95 % CI) for the serum one-carbon metabolites and B-vitamins. The changes in serum one-carbon metabolites and B-vitamins concentrations were calculated as ratios by dividing the concentration at 60 and 120 min by the fasting concentration. The *t* test was used to test if the ratios at each follow-up were different from 1, and paired *t* test was used to test if the ratios in the postprandial samples were different from each other. Genders were compared using independent samples *t* test. All results from T tests were Benjamini-Hochberg adjusted, and results with *P* < 0·05 were considered statistically significant. All statistical tests were performed using R version 4.0.3 (http://www.r-project.org).

## Results

### Description of the standardised breakfast

The contents of vitamins B_2_, B_3_, B_6_, B_9_ and B_12_, total choline, betaine, methionine, serine and glycine in the standardised breakfast are presented in Table 1. Table 1 also presents the RDA for our study participants based on Nordic Nutrition Recommendations^(33)^, and recommendations from the European Food Safety Authority^(34)^ and WHO/FAO/United Nations University^(35)^ and shows that the standardised breakfast contributed with relatively low nutrient amounts compared with the RDAs.

### Fasting serum and urine concentrations of one-carbon metabolites, and serum B-vitamins

The concentrations of metabolites and relevant B-vitamins are presented for the total study population and with separate values for men and women in fasting serum (Table 2) and morning urine (shown relative to creatinine, Table 3). The serum concentration of nicotinic acid was below level of detection in all samples. For most analytes in serum and urine, the concentrations were similar between the genders; however, the serum concentrations of methionine, betaine, sarcosine, trimethyllysine and 3-methylhistidine were highest in men. In urine, the only differences between the genders were the higher concentrations of asymmetric and symmetric dimethyl arginine in women.

**Table 2.** Fasting serum concentrations of metabolites and B-vitamins related to the one-carbon metabolism (Mean values and 95 % confidence intervals)

	Total (*n* 63)		Men (*n* 27)		Women (*n* 36)		*P* sex*
	Mean	95 % CI	Mean	95 % CI	Mean	95 % CI	
Metabolites							
Total homocysteine (µmol/l)	10·0	9·51, 10·6	10·5	9·69, 11·3	9·69	9·00, 10·4	0·31
Methionine (µmol/l)	28·0	27·0, 29·1	30·0	28·4, 31·6	26·6	25·4, 27·8	6·0 × 10^−3^
Choline (µmol/l)	9·92	9·42, 10·5	10·4	9·53, 11·3	9·59	8·97, 10·3	0·21
Betaine (µmol/l)	30·0	27·9, 32·3	34·0	31·2, 37·0	27·3	24·6, 30·4	0·0060
Dimethylglycine (µmol/l)	3·23	2·99, 3·49	3·39	3·00, 3·83	3·11	2·81, 3·45	0·52
Sarcosine (µmol/l)	1·17	1·08, 1·26	1·29	1·15, 1·45	1·07	0·977, 1·18	0·046
Glycine (µmol/l)	249	236, 263	234	223, 246	262	240, 286	0·14
Serine (µmol/l)	133	128, 138	128	121, 136	137	130, 144	0·22
Total cysteine (µmol/l)	299	292, 306	301	293, 311	296	286, 307	0·63
Cystathionine (µmol/l)	0·253	0·222, 0·287	0·266	0·212, 0·333	0·243	0·209, 0·282	0·96
Vitamins							
Flavin mononucleotide (vitamin B_2_, nmol/l)	8·71	7·86, 9·66	9·21	7·85, 10·8	8·33	7·24, 9·58	0·52
Riboflavin (vitamin B_2_, nmol/l)	15·6	13·7, 17·9	16·2	12·7, 20·6	15·2	12·9, 17·8	0·63
Nicotinamide (vitamin B_3_, nmol/l)	220	202, 241	223	192, 258	219	195, 246	0·96
N^1^-methylnicotinamide (vitamin B_3_, nmol/l)	120	107, 134	127	107, 152	114	97·7, 134	0·75
Pyridoxal 5’-phosphate (vitamin B_6_, nmol/l)	39·2	34·8, 44·1	43·5	36·0, 52·6	36·0	30·9, 41·9	0·30
Folate (vitamin B_9_, nmol/l)	18·8	16·6, 21·3	16·7	14·0, 19·9	20·6	17·2, 24·6	0·22
Cobalamin (vitamin B_12_, pmol/l)	303	281, 326	318	287, 352	292	261, 325	0·61
Methylated amino acids							
Asymmetric dimethylarginine (µmol/l)	0·586	0·563, 0·610	0·575	0·537, 0·616	0·594	0·565, 0·625	0·80
Symmetric dimethylarginine (µmol/l)	0·574	0·547, 0·602	0·599	0·563, 0·638	0·555	0·518, 0·594	0·16
Trimethyllysine (µmol/l)	0·667	0·594, 0·749	0·840	0·679, 1·04	0·558	0·509, 0·613	0·012
1-methylhistidine (µmol/l)	4·45	3·50, 5·65	4·31	2·83, 6·57	4·56	3·40, 6·10	0·96
3-methylhistidine (µmol/l)	4·38	4·11, 4·66	4·81	4·39, 5·26	4·07	3·75, 4·42	0·035

*Men and women were compared using independent samples *t* test.

*P* < 0·05 was considered significant.

**Table 3.** Urine concentrations (shown relative to creatinine concentration) of metabolites involved in the one-carbon metabolism (Mean values and 95 % confidence intervals)

Concentrations (µmol/mmol creatinine)	Total (*n* 63)		Men (*n* 27)		Women (*n* 36)		*P* sex*
	Mean	95 % CI	Mean	95 % CI	Mean	95 % CI	
Total homocysteine	0·227	0·193, 0·268	0·202	0·161, 0·253	0·251	0·198, 0·319	0·31
Methionine	0·709	0·632, 0·795	0·706	0·622, 0·800	0·711	0·589, 0·859	1·00
Choline	1·75	1·57, 1·96	1·53	1·29, 1·82	1·96	1·70, 2·27	0·10
Betaine	4·80	3·72, 6·19	5·40	4·05, 7·21	4·35	2·89, 6·55	0·53
Dimethylglycine	2·53	2·10, 3·04	2·41	1·79, 3·23	2·63	2·05, 3·37	0·83
Sarcosine	0·124	0·107, 0·142	0·121	0·096, 0·153	0·126	0·105, 0·151	0·92
Glycine	74·5	65·1, 85·3	64·2	54·8, 75·3	84·3	68·5, 104	0·12
Serine	21·6	19·8, 23·5	19·7	17·3, 22·4	23·3	20·9, 26·1	0·13
Total cysteine	8·61	7·48, 9·92	7·54	6·26, 9·09	9·62	7·82, 11·8	0·22
Cystathionine	2·19	1·80, 2·68	2·19	1·60, 3·00	2·19	1·67, 2·87	1·00
Asymmetric dimethylarginine	1·99	1·88, 2·10	1·72	1·60, 1·85	2·24	2·11, 2·38	7·5 × 10^−6^
Symmetric dimethylarginine	1·82	1·74, 1·90	1·64	1·54, 1·74	1·98	1·89, 2·08	3·1 × 10^−5^
Trimethyllysine	8·33	7·38, 9·40	9·23	7·45, 11·4	7·65	6·67, 8·76	0·27
1-methylhistidine	38·4	30·2, 48·9	33·3	22·7, 48·8	43·3	31·5, 59·6	0·44
3-methylhistidine	30·2	28·2, 32·2	31·9	29·1, 35·1	28·8	26·1, 31·6	0·27

*Men and women were compared using independent samples *t* test.

*P* < 0·05 was considered significant.

Most participants had a serum concentration of folate > 10 nmol/l and vitamin B_12_ > 175 pmol/l. Five participants (four men) had insufficient serum folate concentration, and for three of these (all men), the homocysteine concentration was above the measured median of 10·0 µmol/l. Two participants (both women) had vitamin B_12_ concentration below the reference range, and both had homocysteine concentration >10·0 µmol/l.

### Changes in serum concentrations of metabolites and B-vitamins following a light breakfast

**Fig. 1.**
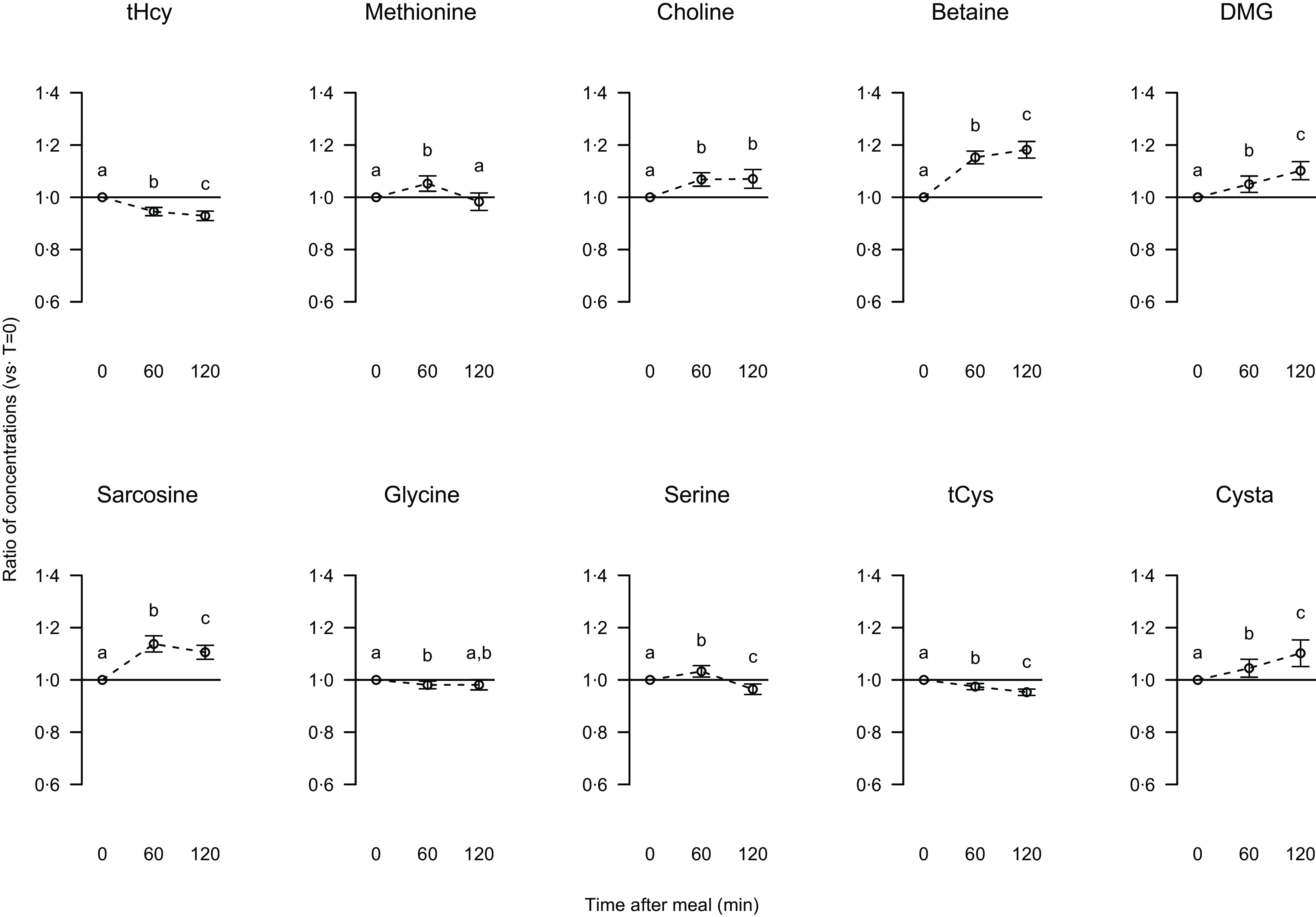
Relative changes from fasting to postprandial serum concentrations of total homocysteine (tHcy), methionine, choline, betaine, dimethylglycine (DMG), sarcosine, glycine, serine, total cysteine (tCys) and cystathionine (Cysta). Data are presented as ratios with 5, 95 % CI for 63 participants. Different letters indicate significant differences at time points (0, 60, 120 min); *P* < 0·05 was considered significant.

The two measured metabolites in the methionine-homocysteine cycle were affected by intake of the breakfast; the serum concentration of total homocysteine was decreased by 6 and 7 % after 60 and 120 min, respectively, whereas the methionine concentration showed an initial 7% increase followed by a decrease to a concentration similar to that in the fasting state (Fig. 1). The postprandial response in total homocysteine concentration was similar between the genders, whereas the ratio of methionine concentration at 120 min relative to fasting concentration was lower in men compared with women (*P* 0·027, data not presented).

The serum concentrations of choline and the choline oxidation pathway metabolites, i.e. betaine, dimethylglycine and sarcosine, were increased after breakfast intake (Fig. 1). After 60 min, the increase was 6 % for choline, 14 % for betaine, 4 % for DMG and 13 % for sarcosine. The increase in betaine concentrations was more pronounced in women compared with men after both 60 and 120 min (*P* values 0·032 and 0·0059, respectively, data not presented). The increase in sarcosine concentration after 120 min was most prominent in women (*P* 0·027), with no differences between the genders for choline and dimethylglycine concentrations (data not presented). The glycine concentration was decreased by 2 % after 60 min (strongest decrease in men, *P* 0·032, data not presented) and was similar to fasting concentration after 120 min (Fig. 1). The serine concentration was first increased by 3 % followed by a decrease to 4 % below fasting concentration (Fig. 1), with no differences between the genders (data not presented).

The transsulfuration pathway interconverts homocysteine and cysteine via the intermediate cystathionine, and the decline in serum homocysteine concentration after breakfast intake was accompanied by lower total cysteine concentration (reduced by 3 and 5 % after 60 and 120 min, respectively); however, the concentration of the intermediate cystathionine was increased postprandially by 4 % after 60 min and further by 8 % after 120 min (Fig. 1). The changes in total cysteine and cystathionine concentrations were similar between the genders (data not presented).

**Fig. 2.**
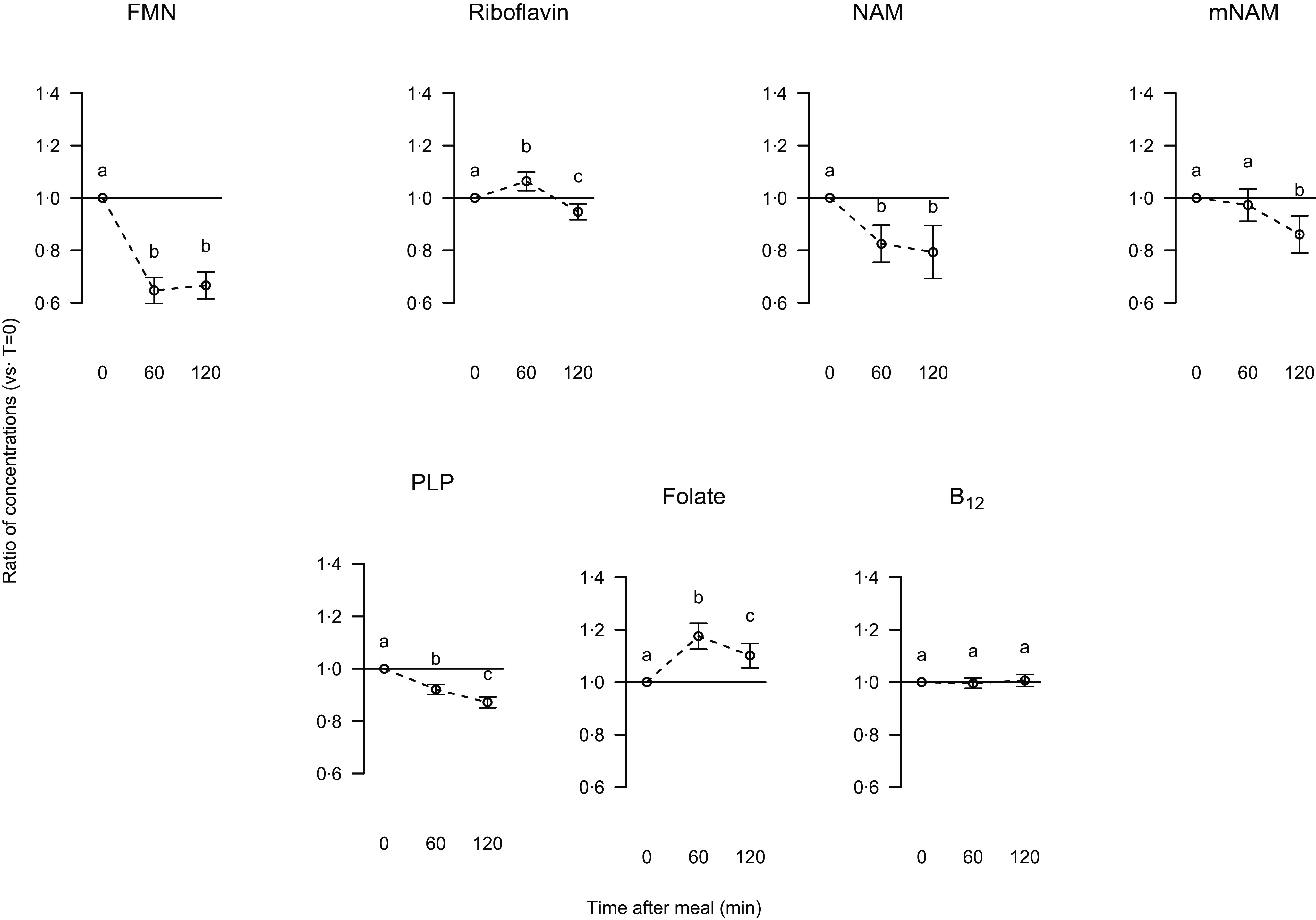
Relative changes from fasting to postprandial serum concentrations of flavin mononucleotide (FMN), riboflavin, nicotinamide, N^1^-methylnicotinamide, pyridoxal 5’-phosphate (PLP), folate and cobalamin (B_12_). Data are presented as ratios with 5, 95 % CI for 63 participants. Different letters indicate significant differences at time points (0, 60, 120 min); *P* < 0·05 was considered significant.

The serum concentrations of flavin mononucleotide, nicotinamide, N^1^-methylnicotinamide and pyridoxal 5’-phosphate were reduced 120 min postprandially, with a decrease of 36, 27, 18 and 13 %, respectively, compared with fasting concentrations. The riboflavin concentration was increased by 6 % after 60 min followed by a reduction to a concentration below fasting concentration (Fig. 2). The concentration of folate was increased by 16 % after 60 min and by 8 % 120 min postprandially, while the cobalamin serum concentration was not affected by breakfast intake (Fig. 2). The decrease in N^1^-methylnicotinamide concentration 60 and 120 min after breakfast intake was more pronounced in men when compared with women (*P* values 0·0033 and 0·026), and the reduction in flavin mononucleotide concentration was more pronounced in women compared with men after 60 min (*P* 0·036) but was similar between genders after 120 min. The postprandial changes in concentrations of the other measured B-vitamins and their derivatives were similar between the genders (data not presented).

### Changes in serum concentrations of methylated amino acids

**Fig. 3.**
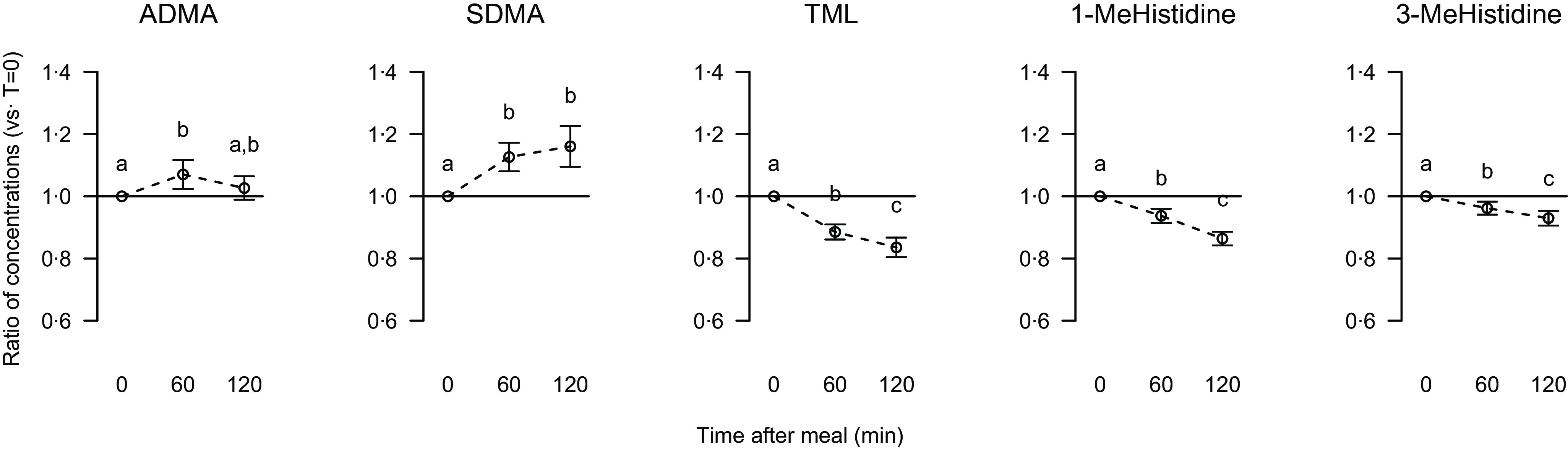
Relative changes from fasting to postprandial serum concentrations of asymmetric dimethylarginine (ADMA), symmetric dimethylarginine (SDMA), trimethyllysine (TML), 1-methylhistidine (1-MeHistidine) and 3-methylhistidine (3-MeHistidine). Data are presented as ratios with 5, 95 % CI for 63 participants. Different letters indicate significant differences at time points (0, 60, 120 min); *P* < 0·05 was considered significant.

Following breakfast intake, the serum concentrations of asymmetric dimethylarginine and symmetric dimethylarginine were increased by 6 and 11 %, respectively, after 60 min, and after 120 min the concentration of asymmetric dimethylarginine was reduced to fasting concentration, whereas concentration of symmetric dimethylarginine remained elevated (Fig. 3). Serum concentrations of trimethyllysine, 1-methylhistidine and 3-methylhistidine were reduced postprandially, and concentrations were 18, 16 and 8 % lower, respectively, after 120 min when compared with fasting concentrations. We observed no differences between the genders for fasting and postprandial concentrations of asymmetric dimethylarginine, symmetric dimethylarginine, trimethyllysine, 1-methylhistidine and 3-mehylhistidine (data not presented).

## Discussion

In this study, we present evidence that the serum concentrations of several metabolites and B-vitamins related to the one-carbon metabolism are affected by a light meal. The serum concentrations of total homocysteine, total cysteine, flavin mononucleotide, nicotinamide and pyridoxal 5’-phosphate were significantly decreased, and the concentrations of choline, betaine, dimethylglycine, sarcosine, cystathionine and folate were significantly increased following breakfast intake. The standardised breakfast contained relatively low amounts of vitamins B_2_, B_3_, B_6_, B_9_ and B_12_ and of choline, betaine and serine, with methionine and glycine below level of detection, suggesting that some of the observed changes may reflect altered metabolic control as the participants’ metabolic status changed from catabolic to anabolic, and not solely the availability of nutrients. We also present evidence that the one-carbon metabolism may be differently affected postprandially in men and women.

We observed a marked increase in serum concentrations of several metabolites in the choline oxidation pathway after breakfast intake. The serum concentrations of both choline and its oxidised product betaine (a methyl donor) and the further demethylated metabolites, dimethylglycine and sarcosine, were increased after breakfast. Choline is obtained from the diet, but the de novo synthesis from phosphatidylethanolamine (via phosphatidylcholine) in liver, using S-adenosylmethionine as a methyl donor, is also a significant source of choline^(36)^. The elevated concentrations of the upstream metabolites in the choline pathways, i.e. choline, betaine, dimethylglycine and sarcosine, observed postprandially might be a consequence of absorption of choline and/or betaine present in the meal. Although contents of choline and betaine in the breakfast were low, a simplified calculation reveals that a median bodyweight of 99·7 kg in our participants and using the factor of 70 ml blood/kg bodyweight (although even lower in adults with overweight or obesity/kg bodyweight^(37)^) gives a median blood volume of less than 7 litres. Thus, and without taking into account the absorption and distribution, the intake of the breakfast meal containing 53·8 mg choline and 30·0 mg betaine may be sufficient to bring about the observed increases in serum concentrations of choline and betaine of 6 and 14 %, respectively, seen 60 min after breakfast in the present study.

Increased availability of choline, and thereby of betaine, may promote betaine-dependent remethylation of homocysteine to methionine in the liver. In addition, an increased serum folate concentration, mainly in the form of 5-methyltetrahydrofolate, may favour increased 5-methyltetrahydrofolate-dependent remethylation of homocysteine to methionine. Increased flux through either pathway may explain the observation of lower homocysteine concentration after consumption of the breakfast meal. The increased methionine concentration after 60 min combined with the reduced homocysteine concentration after breakfast intake may be a result of increased remethylation of homocysteine. However, we cannot rule out the possibility that an intake of methionine from the breakfast may have been sufficient to significantly increase the serum methionine concentration after 60 min. Calculations using data from the USDA database^(38)^ suggest that the methionine content in the breakfast is in the order of around 0·22 g/serving, which is considerably lower than the level of detection for our analyses corresponding to 0·44 g methionine per breakfast serving. Using the same formulas for calculation as for choline and betaine (above), and without taking into account the absorption and distribution, an intake of 0·22 g methionine may in theory be sufficient to induce the 5 % increase in serum methionine concentration seen after 60 min in our study, followed by a reduction after 120 min. This result, together with the knowledge that the methionine concentration peaks after 1 h during the methionine loading test (as demonstrated in several papers, including^(39)^), indicates that the methionine content, although low, in the breakfast may have contributed to the observed increase in serum methionine concentration postprandially. After the initial increase, the methionine concentration was reduced to a concentration comparable to the fasting concentration at 120 min, suggesting that methionine was recycled to homocysteine via S-adenosylmethionine and S-adenosylhomocysteine.

Studies in cultured liver cells present evidence that remethylation of homocysteine is stimulated by insulin and glucose^(21)^. Thus, the increase in insulin and glucose concentrations after breakfast intake, as we have previously published from this trial^(23)^, may contribute to the lower homocysteine and higher methionine concentrations observed postprandially. The serum glucose concentration was significantly higher after 60 min when compared with 120 min and corresponds nicely with the highest methionine concentration after 60 min. Another possible explanation for the lower postprandial homocysteine concentration is an increased conversion of homocysteine to cystathionine through the transsulfuration pathway, which is supported by the increased cystathionine concentration postprandially.

The post-translational methylation of amino acids in proteins is mainly catalysed by S-adenosylmethionine-dependent methyltransferases. Thus, a higher rate of folate-dependent homocysteine remethylation to methionine after food intake may increase the availability of methyl groups delivered by S-adenosylmethionine. Intake of choline and betaine, although found in low amounts in the breakfast, may further contribute to the one-carbon pool. In the present study, we quantified only a few methylated forms of amino acids, but we did not observe a consistent increase in all measured methylated amino acids after breakfast intake. This is most likely due to the different sources of both methylated and non-methylated amino acids, which may originate from the diet or the endogenous body proteins, or both. The synthesis of asymmetric dimethylarginine and symmetric dimethylarginine from arginine residues in proteins is catalysed by S-adenosylmethionine-dependent methyltransferases. Assuming that proteolysis of proteins with methylated arginine residues is low in the anabolic state immediately after breakfast, the elevated concentrations of asymmetric dimethylarginine and symmetric dimethylarginine may reflect their reduced renal clearance or metabolism. The serum concentrations of N^1^-methylnicotinamide, trimethyllysine, 1-methylhistidine and 3-methylhistidine were, however, reduced after breakfast intake. N^1^-methylnicotinamide is produced from nicotinamide using S-adenosylmethionine as methyl donor. The observed postprandial reduction in N^1^-methylnicotinamide may suggest increased metabolism of nicotinamide, which is a precursor for the cofactor nicotinamide adenine dinucleotide, and is involved in choline oxidation pathway. For trimethyllysine and 3-methylhistidine, which are found in, e.g. myosin^(40)^, the lower postprandial serum concentration may be due to lower muscle protein proteolysis in response to increased insulin concentration. A decreased proteolysis cannot explain the reduced 1-methylhistidine concentration since this modified amino acid is neither produced nor found in human muscles^(41)^.

The active forms of vitamins B_2_, B_3_, B_6_ and B_12_ are important cofactors in the transfer of one-carbon units and in the transsulfuration pathway. The standardised breakfast contained very low amounts of these vitamins relative to the recommended daily allowances. In line with this, the serum concentrations of these vitamins were not increased after breakfast intake, with the exception of the 6 % increase in riboflavin after 60 min. Flavin mononucleotide, pyridoxal 5’-phosphate, nicotinamide and N^1^-methylnicotinamide showed lower serum concentration after breakfast intake, indicating an increased utilisation, with no change in vitamin B_12_ concentration postprandially. This underscores the importance of controlling for food intake in studies involving the one-carbon metabolism, even in settings where the consumed food has low contents of nutrients including B-vitamins.

In the present study, we observed higher fasting serum concentrations of methionine, sarcosine and betaine in men when compared with women, which is partly in line with observations in a larger study in cancer-free older adults^(42)^. The higher fasting serum concentrations of trimethyllysine and 3-methylhistidine observed in our male participants may be a consequence of the larger muscle mass in men, since both methylated amino acids are found in muscle proteins^(40)^. Although we observed no differences between the genders for asymmetric and symmetric dimethylarginine in fasting serum, the urine concentrations (relative to creatinine) of these dimethylarginines were markedly lower in men. The lower urine concentrations of asymmetric and symmetric dimethylarginine in men may indicate lower synthesis from arginine, possibly caused by an inhibiting effect of testosterone on the involved methyltransferases^(43)^, since our participants had normal kidney function as evidenced by serum creatinine concentration and urine albumin creatinine ratio within normal ranges^(25)^. For the majority of metabolites and B-vitamins related to the one-carbon metabolism that were investigated in the present study, their relative changes in serum concentrations after breakfast were comparable between men and women. We did, however, observe some gender differences that should be mentioned. Some of the differences between the genders may be difficult to explain, such as the more prominent postprandial increases in serum concentrations of betaine and sarcosine seen in women, combined with no differences in choline and dimethylglycine between the genders. Another striking difference between the genders worth commenting was the markedly larger reduction in N^1^-methylnicotinamide concentration seen in men compared with women after breakfast. The breakdown of N^1^-methylnicotinamide by N^1^-methylnicotinamide oxidase is stimulated by testosterone, and the activity of the enzyme is several fold higher in male compared with female mice^(44)^. This may, at least in part, explain the distinct effect on N^1^-methylnicotinamide concentration in men after breakfast intake.

The present study has some strengths and limitations. The strengths of the study include that all participants consumed a well-characterised light meal following an over-night fast, and the sample size was relatively large and consisted of both men and women. A broad array of metabolites and B-vitamins related to one-carbon metabolic pathways were measured. The serum and urine samples were analysed using established methods with high precision. We strictly followed a defined protocol for pre-analytical handling of samples, and samples were thawed for the first time for these analyses and showed no signs of degradation. The limitations include the generalisability of the findings for other populations such as those with normal bodyweight, other age groups and patients with established metabolic disturbances or diseases and the use of meals with other ingredients and serving sizes.

### Conclusion

Our findings demonstrate that the consumption of a light breakfast high in carbohydrates but with low nutrient content was sufficient to induce changes in circulating concentrations of several metabolites and B-vitamins related to the one-carbon metabolism. The one-carbon metabolism may be differently affected postprandially in men and women, since we observed differences in changes from the fasting to the postprandial state for some of the measured metabolites between the genders. Our novel findings underline the importance of having information regarding the prandial state of the study participants or patients in epidemiological and intervention studies when exploring metabolites and B-vitamins related to the one-carbon metabolism.
